# A comparative study of the efficacy of intra-articular injection of different drugs in the treatment of mild to moderate knee osteoarthritis: A network meta-analysis

**DOI:** 10.1097/MD.0000000000033339

**Published:** 2023-03-24

**Authors:** Yuan Xue, Xuan Wang, Xiuming Wang, Li Huang, Aina Yao, Yan Xue

**Affiliations:** a Shanxi University of Chinese Medicine, Taiyuan, Shanxi, China; b Shanxi Acupuncture and Moxibustion Hospital/Shanxi Institute of Acupuncture and Moxibustion, Taiyuan, Shanxi, China; c Northwest Women and Children’s Hospital, Xian, Shaanxi, China.

**Keywords:** durgs, injections, intra-articular, knee, network meta-analysis, osteoarthritis

## Abstract

Knee osteoarthritis (KOA) has a high clinical prevalence and frequently interferes with patients normal lives. In KOA patients, evidence suggests that intra-articular (IA) injection improves joint function and decreases discomfort. Several IA injection treatments are used in daily practice to improve symptomatic control of knee osteoarthritis, but their efficacy is frequently disputed.

**Methods:**

This network meta-analysis compares the efficacy of different IA injections for mild to moderate knee osteoarthritis. Seven databases (PubMed, EMBASE, Web of Science, Cochrane Library, China Biology Medicine disc, WanFang, and China National Knowledge Infrastructure) were searched for randomized controlled trials published up to and including December 20, 2021, and final follow up indicators were used. Visual analogue scale (VAS) score and The Western Ontario and McMaster Universities (WOMAC) Osteoarthritis Index (WOMAC) score change from baseline were the primary outcomes. We used the Cochrane risk of bias tool to assess the quality and risks of biases of papers. We calculated the weighted mean difference (WMD) and 95% confidence interval (CI) for each outcome. State (Version 15.1, Texas, USA) and SPSS (Version 20, Chicago, USA) was used in all statistical analyses, and Review Manager (version 5.4) was used in assessing the risks of biases.

**Results:**

Our study included 16 randomized controlled trials with a total of 1652 patients. platelet-rich plasma (PRP) IA injection therapy had the highest likelihood of being the best intervention in reducing WOMAC pain (surface under the cumulative ranking area [SUCRA] 84.7%), stiffness (SUCRA 95.1%), and function (SUCRA 98.5%) scores, according to the SUCRA. The best measures for lowering the WOMAC total and VAS scores were IA injection platelet-rich plasma-derived growth factor (SUCRA 84.9%) and hyaluronic acid and platelet-rich plasma (SUCRA 84.9%). In the VAS score group, PRP outperformed hyaluronic acid (HA) (WMD 1.3, 95% CI 0.55–2.55) and corticosteroids (CS) (WMD 4.85, 95% CI 4.02–5.08), according to the forest map results. PRP also outperformed CS (WMD 14.76, 95% CI 12.11–17.41), ozone (WMD 9.16, 95% CI 6.89–11.43), and PRP + HA (WMD 2.18, 95% CI 0.55–3.81) in the WOMAC total score group. Furthermore, PRP outperforms other drugs in terms of reducing WOMAC function, stiffness, and function score.

**Conclusion:**

In patients with mild to moderate KOA, IA injection PRP outperformed IA injection ozone, HA, CS, platelet-rich plasma-derived growth factor, and hyaluronic acid and platelet-rich plasma in terms of pain, stiffness, and dysfunction.

## 1. Introduction

Osteoarthritis (OA) is a degenerative joint disease characterized by the slow progressive destruction of articular cartilage, with knee osteoarthritis (KOA) being primarily caused by wear and tear due to aseptic articular cartilage inflammation.^[1]^ According to epidemiological survey results, approximately 250 million people worldwide suffer from knee osteoarthritis. As the general population ages, the prevalence of KOA rises, and KOA has become a major public health concern worldwide.^[2]^ KOA patients frequently experience knee pain, limited mobility, and in severe cases, disability, all of which have a negative impact on their lives.

Conservative therapy is less expensive than surgical therapy and has no risk of surgical complications or postoperative sequelae, so it is preferred by doctors and patients in clinical practice. In comparison to oral medications, surgery, and other treatments, intra-articular (IA) drug injections may be crucial in the management of OA.^[3]^ Oral nonsteroidal anti-inflammatory drugs, glucosamine, and chondroitin sulfate have been shown to be effective for pain relief and functional improvement in a short period of time as a form of conservative therapy, but there is no evidence of improvement in the underlying knee condition at this stage,^[4,5]^ and long-term NSAID use has a high risk of associated side effects, and IA injections have been advocated as an alternative to oral medication for KOA.^[6]^ However, injection therapy involves a variety of drugs, including hyaluronic acid, corticosteroids, platelet-rich plasma, plasma rich in growth factors, ozone, and others. Which drug is the most effective for IA therapy in patients with mild to moderate KOA? Unfortunately, few studies compared these drugs in patients with mild to moderate KOA.

A large number of related meta-analyses have been published in recent years, but most of them^[6–8]^ tend to compare 1 drug with another, or to compare controls applied with 1 drug with controls with no treatment, making it impossible to compare the efficacy of multiple control groups comprehensively. Some previously published network meta-analysis articles^[9,10]^ compared the therapeutic effects of various IA injections on KOA, but plasma rich in growth factors, ozone, and hyaluronic acid and platelet-rich plasma (HA + PRP) were not included, and no specific target drugs for mild to moderate KOA were identified. Although previously published meta-analysis articles compared the therapeutic efficacy of various IA injection therapies for KOA, few studies have been conducted on growth factor-rich plasma, ozone, and HA + PRP. Thus, we considered the need for the current study in order to address these issues.

## 2. Materials and Methods

### 2.1. Search strategy

Two independent reviewers (Yuan and Xuan) searched and identified relevant studies in the following databases in December 2021: PubMed, EMBASE, Web of Science, Cochrane Library, China Biology Medicine disc, WanFang, and China National Knowledge Infrastructure. The following keywords and synonyms were used in tandem: Ozone Therapy; Hyaluronic Acid; Platelet-Rich Plasma; Plasma Rich Growth Factor; Corticosteroids; Randomized Controlled Trial. Trials were considered without regard to language. Consider PubMed; the specific retrieval strategy is shown in Table 1. A third author resolved disagreements among reviewers and evaluated all articles.

**Table 1:** Search strategy used in PubMed database.

Number	Search items
#1	Randomized controlled trial [Publication type]
#2	Randomized [Title/abstract]
#3	Placebo [Title/abstract]
#4	OR/#1–#3
#5	osteoarthritis, knee [MeSH terms]
#6	knee osteoarthritis [All fields]
#7	osteoarthritis of the knee [All fields]
#8	KOA [All fields]
#9	Osteoarthritides [All fields]
#10	OR/#5–#9
#11	Hyaluronic acid [MeSH terms]
#12	Hyaluronate sodium OR sodium hyaluronate OR sodium hyaluronic acid OR
#13	Acid hyaluronic OR amo vitrax OR vitrax, amo OR biolon OR etamucine OR hyaluronan OR hyvisc OR luronit OR sodium hyaluronate OR hyaluronate OR sodium OR amvisc OR healon [All fields] OR/#11–#12
#14	Platelet-rich plasma [MeSH terms]
#15	Plasma, platelet-rich OR platelet-rich plasma OR PRP [All fields]
#16	OR/#14–#15
#17	Plasma rich growth factor [MeSH terms]
#18	PRGF [All fields]
#19	OR/#17–#18
#20	Corticosteroids [MeSH terms]
#21	Adrenal cortex hormones OR hormones, adrenal cortex OR
#22	Corticosteroids OR corticosteroid OR corticoids OR corticoid OR adrenal cortex hormone OR cortex hormone, adrenal OR hormone, adrenal cortex [All fields] OR/#20–#21
#23	Ozone therapy [MeSH terms]
#24	Ozone injection OR medicinal ozone OR O_3_ triatomic variety of oxygen OR ozone treatment OR oxygen/ozone therapy OR O_2_–O_3_
#25	OR/#23–#24
#26	OR/#13 OR #16 OR #19 OR #22 OR #25
#27	4 AND #10 AND #26

KOA = knee osteoarthritis, PRGF = platelet-rich plasma-derived growth factor, PRP = platelet-rich plasma.

### 2.2. Study selection

Only randomized controlled trial (RCT) with a high level of evidence were included in this meta-analysis, while expert opinions, conference abstracts, observational studies, and animal studies were excluded. The following were the study inclusion criteria: The study’s subjects were patients aged 18 to 80 years old with mild to moderate KOA (the Kellgren-Lawrence Radiology Scale system score ranged from 1–3), regardless of gender or race; After treatment, visual analogue scale (VAS) and The Western Ontario and McMaster Universities (WOMAC) osteoarthritis index (WOMAC) scores were reported as study outcomes; The intervention consisted of IA injections of platelet-rich plasma (PRP), platelet-rich plasma-derived growth factor (PRGF), hyaluronic acid (HA), ozone, and corticosteroids (CS). These drugs were also included in the comparison group.

### 2.3. Exclusion criteria

We excluded the following: Studies aimed at KOA patients with any combination of knee fractures, meniscus avulsion, tendon injuries, history of severe knee traumas, systemic diseases such as diabetes, immunodeficiency, collagen vascular diseases, history of malignancies, knee infection or active wounds, autoimmune diseases, diseases affecting platelets, and those with major underlying diseases; All studies with no control groups were ruled out; Research lacking primary data, or whose authors could not be contacted and were unavailable even after contacting them. Patients with a history of joint surgery in the previous 6 months, those who had any invasive surgery on the affected joint, as well as those with abnormal blood counts or impaired coagulation tests, were all excluded from this study; Patients taking nonsteroidal anti-inflammatory drugs 2 days before the injection, anticoagulant or antiplatelet drugs 10 days before the injection, knee steroid injection 3 weeks before the test, systemic steroid injection 2 weeks before the experiment, pregnant or lactating women, patients with a history of vasovagal shock, cancer or malignancy, or infections or ulcers in the target knee were not included in this study.

### 2.4. Date extraction

Data extraction was primarily completed by 2 independent reviewers (Yuan, Xuan), who recorded the demographic baseline of the included patients (mean ages, sexes, BMI, and gender ratio), as well as general information from the included papers: authors and years, treatment regimens, and adverse reactions after injection. Data extraction of relevant results includes the VAS and WOMAC score as well.

### 2.5. Methodological quality assessment

Two independent reviewers assessed the quality of the articles (Yuan, Xuan). A third party resolved disagreements between reviewers. The risk of bias summary was completed using the Review Manager 5.4 software (The Nordic Cochrane Collaboration, Copenhagen) on the following aspects: randomization methods, allocation schemes, the use of blinded experiments, data completeness, and study results reporting. The quality of the RCTs was also evaluated by looking at the following factors: Allocation concealment; sequence generation; Outcome evaluation blinding; Selective reporting; Incomplete data reporting; Other biases.

### 2.6. Statistical analysis

The lead author was primarily responsible for the statistical analysis (Yuan). STATA/MP 15.1 was used to perform a network meta-analysis, and RevMan 5.4 was used to assess the quality of the literature. For each outcome, we used a network graph to present all treatment comparisons. The sizes of the dots in the graph represent the sample sizes of the interventions, and the thickness of the lines represents the amount of direct evidence between the 2 interventions in the evidence network. The random effects model was used in the study to calculate the weighted mean difference (WMD) and 95% confidence interval (CI) for each pairwise outcome. The surface under the cumulative ranking curve (SUCRA) was used to rank each indicator for each treatment method, with higher SUCRA probabilities indicating that the treatment has a better chance of being the best. A funnel plot was used to determine whether there was a small sample effect among studies and whether the included studies had a publication bias. Kappa consistency tests were run on the number of articles finally included and excluded by both reviewers (Yuan, Xuan) using SPSS (version 20). This was used to assess the consistency of the results of the literature that had been screened by 2 reviewers and was eventually included in the analysis.

## 3. Results

### 3.1. Search result

**Figure 1.**
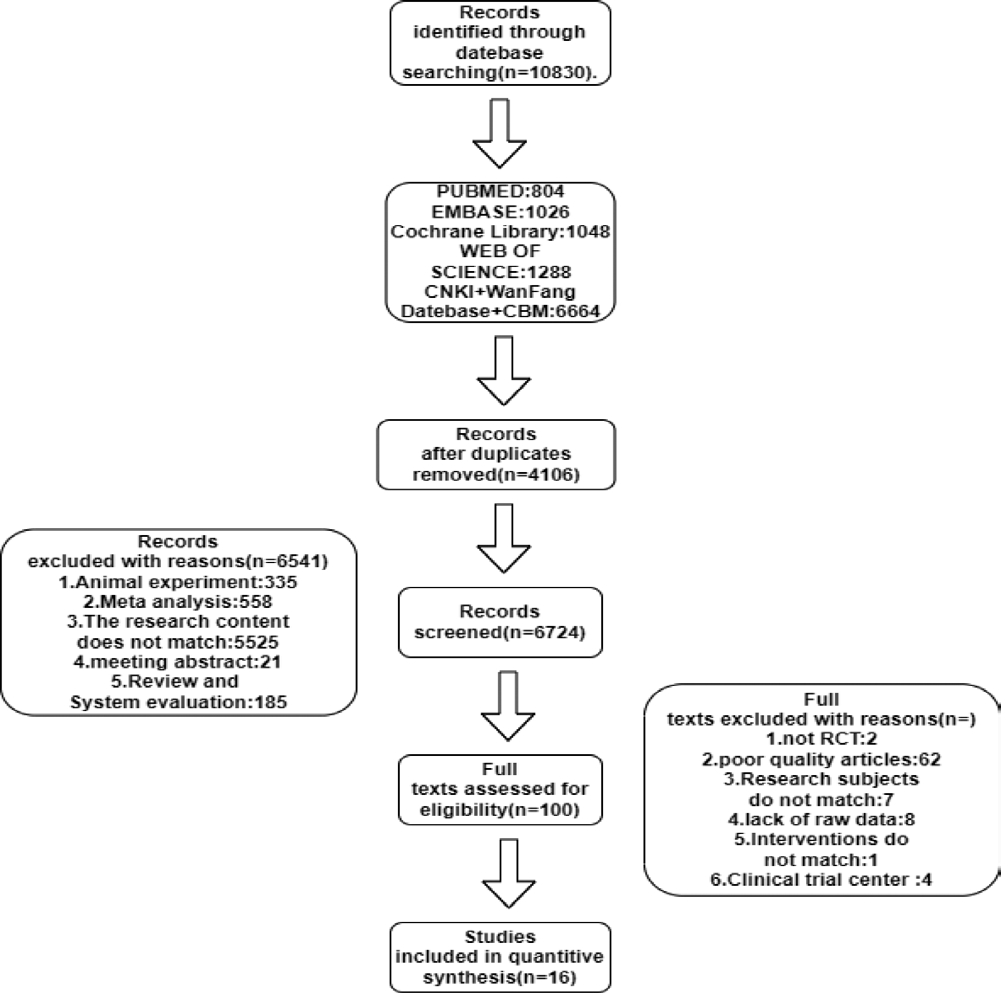
Literature search flowchart.

After searching 4 English databases (PubMed, EMBASE, Web of Science, and the Cochrane Library) and 3 Chinese databases (China Biology Medicine disc, WanFang, and China National Knowledge Infrastructure), the retrieval dates ranged from the databases inception dates to December 2021. Table 2 shows the quantity of literature in each database. In the beginning, 10,830 articles were retrieved and imported into the document management software Note Express (version: 3.5.0.9054). Reviewer Yuan chose to include 16 studies, while reviewer Xuan chose to include 20 (which included all of Yuan literature). Finally, the third author chose to include 16 works of literature. In the end, 16 articles were chosen for analysis (Fig. 1). On how many pieces of literature the 2 reviewers included or excluded; we ran a Kappa consistency test. We imported the data of the number of included and excluded literature for both reviewers into SPSS software and then weighted the cases to form a cross-tabulation after which the number of included and excluded literature for both reviewers were added to the rows and columns respectively, and finally the kappa option was selected to obtain the final results. The findings revealed a *P* value of .000 < .05 and the kappa value of 0.889 > 0.75, showing that the methodological consistency between the 2 reviewers on the inclusion and exclusion of the literature was statistically significant and the agreement was strong.

**Table 2:** Number of documents in each database.

Database	Number of literature
PUBMED	804
EMBASE	1026
Cochrane library	1048
Web of science	1288
CNKI, WanFang, CBMdisc	6664

CBMdisc = China Biology Medicine disc, CNKI = China National Knowledge Infrastructure.

### 3.2. Methodological quality assessment

**Figure 2.**
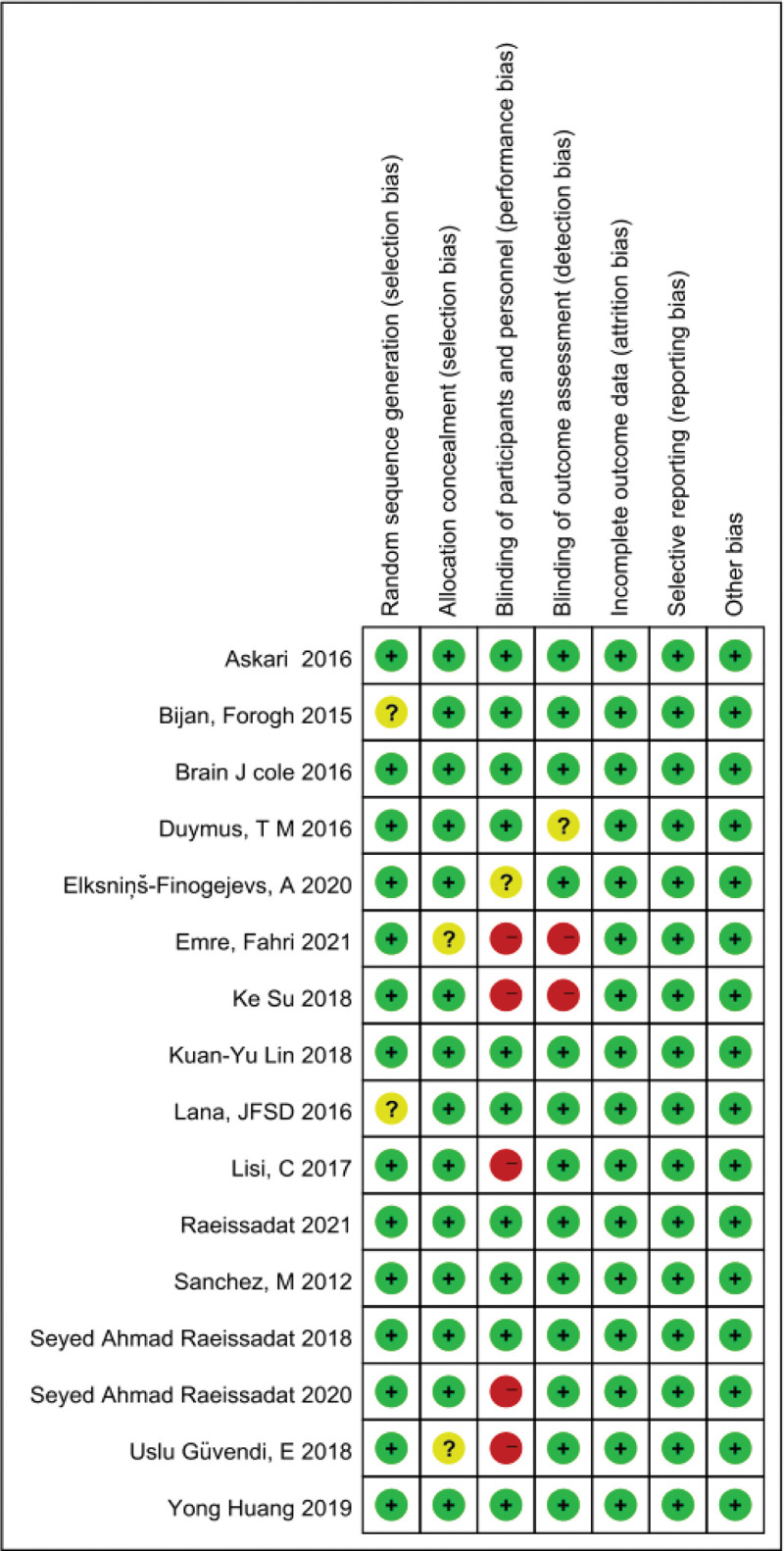
Methodological quality.

This study has the advantage of having a low risk of bias because 87.5% of the studies were blinded, with 68.75% of them being double-blinded. Because of differences in injection product viscosity, the impossibility for blind patients to extract the autologous plasma, and the difficulty for blind researchers to inject PRP and PRGF, the unblinded approach of this type of research can be considered non-high-risk (Emre, Fahri 2021 injection of PRP) (Ke Su 2018 injection of PRP) (Lisi, C 2017 injection of PRGF) (Seyed Ahmad Raeissadat 2020 injection of PRGF). Furthermore, the risk of attrition, reporting, and unidentified biases is low. Therefore, the overall risk of bias is low, and the quality of the article is good (Fig. 2).

### 3.3. Primary outcomes

Primary outcomes included VAS and WOMAC score.

#### 3.3.1. Network map.

**Figure 3.**
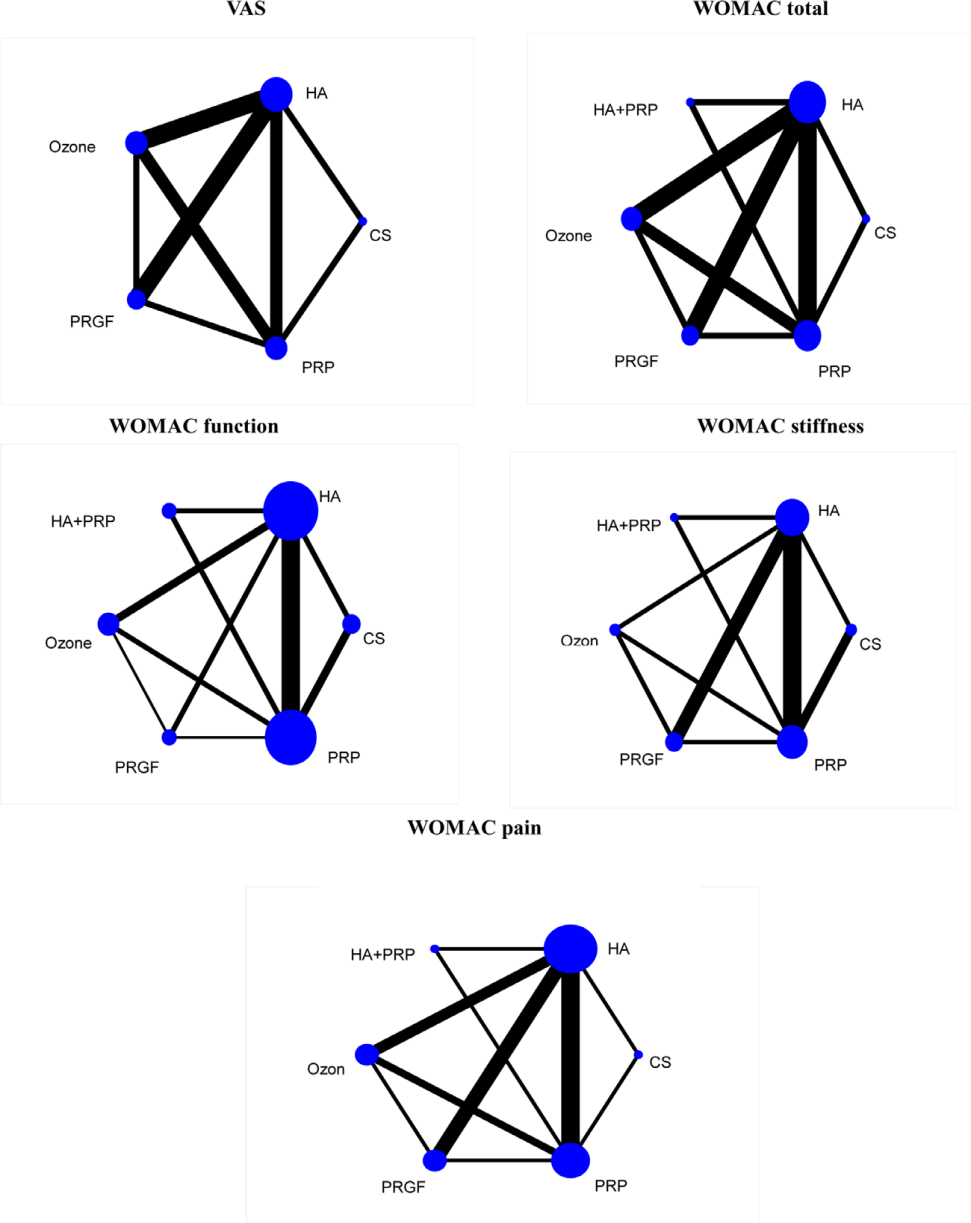
Network of VAS, WOMAC total, WOMAC function, WOMAC stiffness and WOMAC pain. VAS = visual analogue scale, WOMAC = The Western Ontario and McMaster Universities (WOMAC) osteoarthritis index.

Each dot in the network diagram represents an intervention. The larger the dot area, the larger the population of the studied intervention. The line connecting the 2 dots represent a direct comparison to RCT studies of 2 interventions. The thickness of the line represents the number of direct comparisons to RCT studies between 2 interventions (Fig. 3).

#### 3.3.2. VAS score.

**Figure 4.**
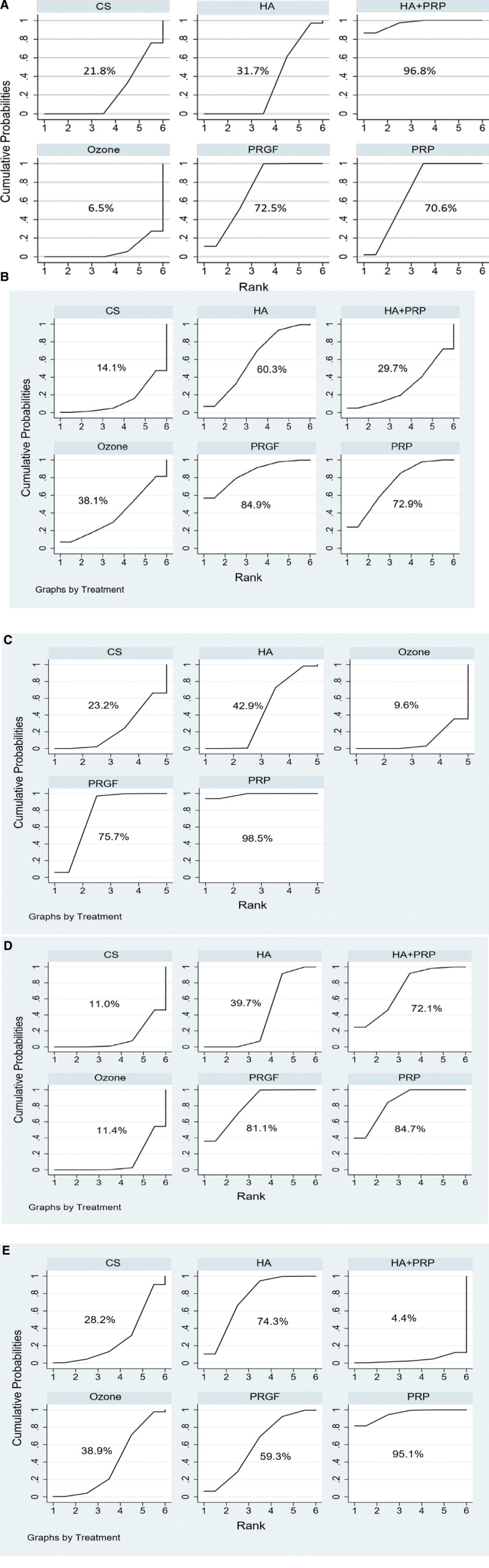
Cumulative probabilities table (A) VAS, (B) WOMAC total, (C) WOMAC function, (D) WOMAC pain, and (E) WOMAC stiffness. VAS = visual analogue scale, WOMAC = The Western Ontario and McMaster Universities (WOMAC) osteoarthritis index.

**Figure 5.**
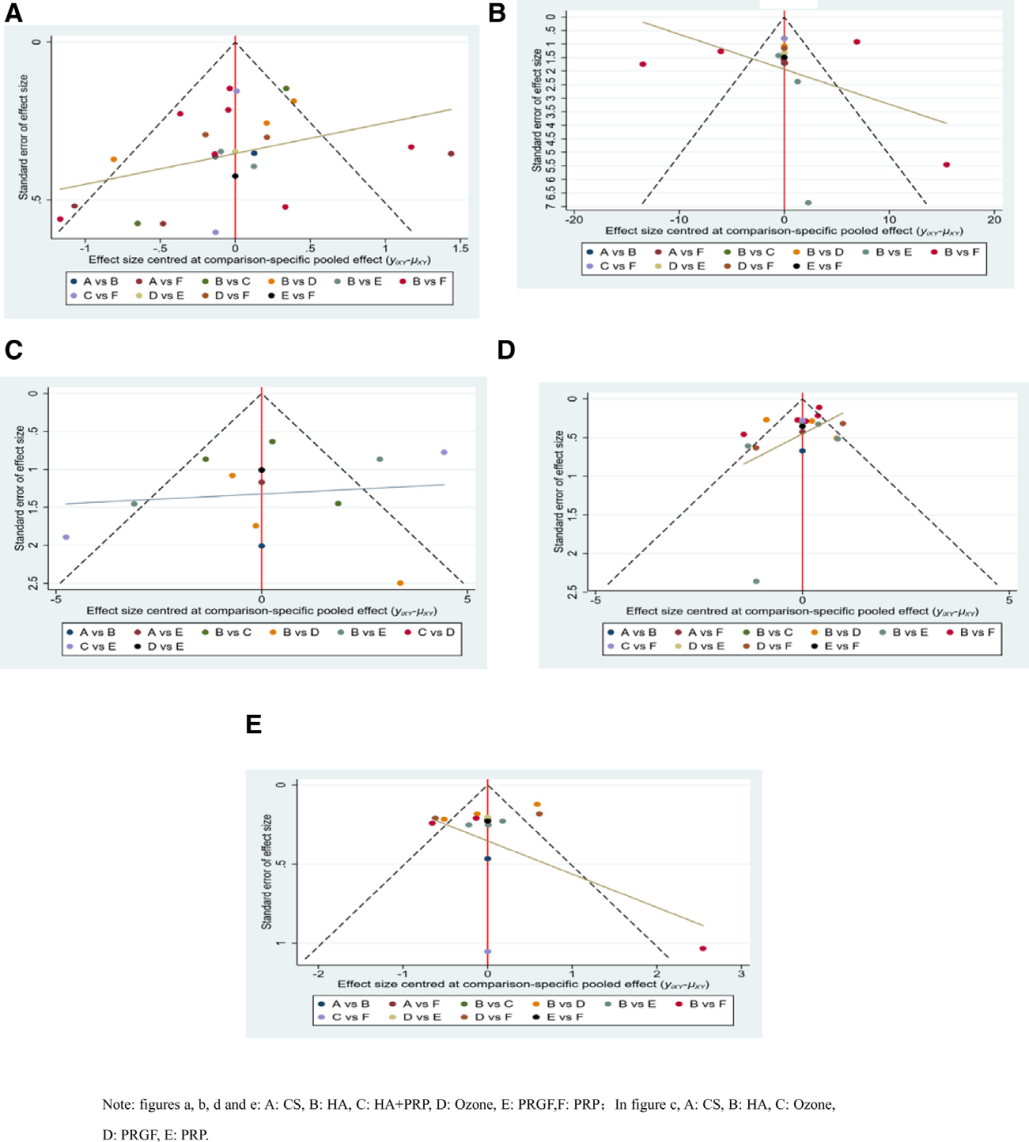
Pairwise comparison of forest plots.

As shown in Figure 4A, the VAS score effects of all drugs were ranked using SUCRA probabilities (96.8%), and HA + PRP had the highest probability (96.8%) of being the best treatment option for reducing VAS score, followed by groups of PRGF, PRP, HA, CS, and ozone, and the cumulative probability SUCRA ranking was: HA + PRP (96.8%) > PRGF (72.5%) > PRP (70.6%) >HA (31.7%) > CS (21.8%) > ozone (6.5%). However, as shown in Figure 5A, there was no significant difference between IA injection HA + PRP versus PRP and PRP versus PRGF. PRP significantly reduced VAS score when compared to other drugs, outperforming HA (WMD 1.3, 95% CI 0.55–2.55) and CS (WMD4.85, 95% CI 4.02–5.08). Furthermore, PRGF outperformed ozone (WMD 2.70, 95% CI 2.01–3.39) and HA + PRP outperformed HA (WMD 1.25, 95% CI 0.66–1.84) and HA outperformed ozone (WMD 1.27, 95% CI 0.10–2.44). Table 3 shows the pairwise comparison.

**Table 3:** VAS score pairwise comparison.

HA + PRP	0.71 (−0.46, 1.88)	0.72 (−0.10, 1.55)	2.22 (1.40, 3.05)	2.37 (1.33, 3.42)	2.69 (1.68, 3.70)
−0.71 (−1.88, 0.46)	PRGF	0.01 (−0.90, 0.93)	1.51 (0.66, 2.37)	1.66 (0.58, 2.75)	1.98 (1.01, 2.95)
−0.72 (−1.55, 0.10)	−0.01 (−0.93, 0.90)	PRP	1.50 (1.05, 1.95)	1.65 (0.95, 2.35)	1.97 (1.27, 2.66)
−2.22 (−3.05, −1.40)	−1.51 (−2.37, −0.66)	−1.50 (−1.95, −1.05)	HA	0.15 (−0.56, 0.86)	0.47 (−0.18, 1.11)
−2.37 (−3.42, −1.33)	−1.66 (−2.75, −0.58)	−1.65 (−2.35, −0.95)	−0.15 (−0.86, 0.56)	CS	0.32 (−0.61, 1.24)
−2.69 (−3.70, −1.68)	−1.98 (−2.95, −1.01)	−1.97 (−2.66, −1.27)	−0.47 (−1.11, 0.18)	−0.32 (−1.24, 0.61)	Ozone

CS = corticosteroids, HA = hyaluronic acid, HA + PRP = hyaluronic acid and platelet-rich plasma, PRGF = platelet-rich plasma-derived growth factor, PRP = platelet-rich plasma, VAS = visual analogue scale.

#### 3.3.3. WOMAC total score.

The PRGF had the highest probability (84.9%) of being the best treatment option for lowering the WOMAC total score, followed by PRP, HA, ozone, HA + PRP, and CS, and the cumulative probability SUCPA ranking was as follows: PRGF (84.9%) > PRP (72.9%) > HA (60.3%) > ozone (38.1%)>HA + PRP (29.7%)>CS (14.1%) (Fig. 4B). When compared to ozone (WMD 9.16, 95% CI 6.89–11.43), CS (WMD 14.76, 95% CI 12.11–17.41), and HA + PRP (WMD 2.18, 95% CI 0.55–3.81), PRP significantly reduced the WOMAC total score (Fig. 5B). PRGF outperformed ozone (WMD9.18, 95% CI 6.58–11.78) but did not differ significantly from PPR. Table 4 shows the outcome of the pairwise comparison.

**Table 4:** WOMAC total score pairwise comparison table.

PRGF	2.86 (−9.02, 14.74)	4.73 (−5.39, 14.85)	9.32 (−6.02, 24.67)	11.37 (−6.37, 29.11)	15.25 (−0.21, 30.71)
−2.86 (−14.74, 9.02)	PRP	1.87 (−6.59, 10.32)	6.46 (−8.52, 21.45)	8.51 (−6.61, 23.63)	12.39 (1.01, 23.77)
−4.73 (−14.85, 5.39)	−1.87 (−10.32, 6.59)	HA	4.59 (−9.96, 19.14)	6.64 (−8.48, 21.76)	10.52 (−2.13, 23.17)
−9.32 (−24.67, 6.02)	−6.46 (−21.45, 8.52)	−4.59 (−19.14, 9.96)	Ozone	2.05 (−18.23, 22.32)	5.93 (−12.23, 24.09)
−11.37 (−29.11, 6.37)	−8.51 (−23.63, 6.61)	−6.64 (−21.76, 8.48)	−2.05 (−22.32, 18.23)	HA + PRP	3.88 (−14.49, 22.24)
−15.25 (−30.71, 0.21)	−12.39 (−23.77, −1.01)	−10.52 (−23.17, 2.13)	−5.93 (−24.09, 12.23)	−3.88 (−22.24, 14.49)	CS

CS = corticosteroids, HA = hyaluronic acid, HA + PRP = hyaluronic acid and platelet-rich plasma, PRGF = platelet-rich plasma-derived growth factor, PRP = platelet-rich plasma, WOMAC = The Western Ontario and McMaster Universities (WOMAC) osteoarthritis index.

#### 3.3.4. WOMAC function, pain, and stiffness.

Based on our findings, PRP is likely to be the most effective treatment for dysfunction, pain, and stiffness in patients with mild to moderate knee osteoarthritis. The cumulative probability SUCPA was ranked as follows: PRP (98.5%) > PRGF (75.7%) > HA (42.9%) > CS (23.2%) > ozone (9.6%), PRP (84.7%) > PRGF (81.1%) > HA + PRP (72.1%) > HA (39.7%)>ozone (11.4%)>CS (11.0%), and PRP (95.1%) > HA (74.3%) > PRGF (59.3%) > ozone (38.9%) > CS (28.2%) > HA + PRP (4.4%). The results of the analysis are shown in Figure 4C, D, and 4E, and the pairwise comparison table is shown in Tables 5, 6 and 7. As shown in Figure 5C, D, and E, PRP outperformed HA (WMD 3.55, 95% CI 0.38–6.72) (WMD 1.28, 95% CI 0.49–2.07), ozone (WMD 10.89, 95% CI 6.97–14.81) (WMD 3.80, 95% CI 2.53–5.07), CS (WMD 11.26, 95% CI 8.97–13.56) (WMD 4.85, 95% CI 4.02–5.68). Furthermore, PRP was more effective in reducing WOMAC stiffness score than PRGF (WMD 0.55, 95% CI 0.1–1), CS (WMD 1.78, 95% CI 1.35–2.21), and ozone (WMD 1.56, 95% CI 1.05–2.07).

**Table 5:** WOMAC function score pairwise comparison table.

PRP	3.54 (−0.97, 8.04)	7.91 (4.30, 11.52)	9.68 (4.95, 14.42)	10.77 (6.75, 14.79)
−3.54 (−8.04, 0.97)	PRGF	4.37 (0.81, 7.94)	6.15 (0.32, 11.98)	7.24 (2.96, 11.51)
−7.91 (−11.52, −4.30)	−4.37 (−7.94, −0.81)	HA	1.78 (−3.15, 6.70)	2.86 (−0.39, 6.12)
−9.68 (−14.42, −4.95)	−6.15 (−11.98, −0.32)	−1.78 (−6.70, 3.15)	CS	1.09 (−4.46, 6.64)
−10.77 (−14.79, −6.75)	−7.24 (−11.51, −2.96)	−2.86 (−6.12, 0.39)	−1.09 (−6.64, 4.46)	Ozone

CS = corticosteroids, HA = hyaluronic acid, PRGF = platelet-rich plasma-derived growth factor, PRP = platelet-rich plasma, WOMAC = The Western Ontario and McMaster Universities (WOMAC) osteoarthritis index.

**Table 6:** WOMAC pain score pairwise comparison table.

PRP	0.11 (−1.32, 1.55)	0.45 (−1.52, 2.42)	1.90 (0.97, 2.82)	3.15 (1.80, 4.50)	3.26 (1.52, 5.00)
−0.11 (−1.55, 1.32)	PRGF	0.34 (−1.96, 2.63)	1.79 (0.55, 3.02)	3.04 (1.45, 4.62)	3.15 (1.04, 5.27)
−0.45 (−2.42, 1.52)	−0.34 (−2.63, 1.96)	HA + PRP	1.45 (−0.52, 3.42)	2.70 (0.44, 4.96)	2.82 (0.26, 5.37)
−1.90 (−2.82, −0.97)	−1.79 (−3.02, −0.55)	−1.45 (−3.42, 0.52)	HA	1.25 (0.04, 2.46)	1.37 (−0.40, 3.14)
−3.15 (−4.50, −1.80)	−3.04 (−4.62, −1.45)	−2.70 (−4.96, −0.44)	−1.25 (−2.46, −0.04)	Ozone	0.12 (−1.97, 2.20)
−3.26 (−5.00, −1.52)	−3.15 (−5.27, −1.04)	−2.82 (−5.37, −0.26)	−1.37 (−3.14, 0.40)	−0.12 (−2.20, 1.97)	CS

CS = corticosteroids, HA = hyaluronic acid, HA + PRP = hyaluronic acid and platelet-rich plasma, PRGF = platelet-rich plasma-derived growth factor, PRP = platelet-rich plasma, WOMAC = The Western Ontario and McMaster Universities (WOMAC) osteoarthritis index.

**Table 7:** WOMAC stiffness score pairwise comparison table.

PRP	0.44 (−0.32, 1.19)	0.63 (−0.28, 1.54)	0.97 (0.16, 1.78)	1.29 (0.26, 2.31)	2.67 (0.57, 4.76)
−0.44 (−1.19, 0.32)	HA	0.19 (−0.50, 0.89)	0.53 (−0.14, 1.21)	0.85 (−0.19, 1.89)	2.23 (0.14, 4.33)
−0.63 (−1.54, 0.28)	−0.19 (−0.89, 0.50)	PRGF	0.34 (−0.52, 1.21)	0.66 (−0.55, 1.86)	2.04 (−0.15, 4.22)
−0.97 (−1.78, −0.16)	−0.53 (−1.21, 0.14)	−0.34 (−1.21, 0.52)	Ozone	0.32 (−0.84, 1.47)	1.70 (−0.46, 3.86)
−1.29 (−2.31, −0.26)	−0.85 (−1.89, 0.19)	−0.66 (−1.86, 0.55)	−0.32 (−1.47, 0.84)	CS	1.38 (−0.89, 3.66)
−2.67 (−4.76, −0.57)	−2.23 (−4.33, −0.14)	−2.04 (−4.22, 0.15)	−1.70 (−3.86, 0.46)	−1.38 (−3.66, 0.89)	HA + PRP

CS = corticosteroids, HA = hyaluronic acid, HA + PRP = hyaluronic acid and platelet-rich plasma, PRGF = platelet-rich plasma-derived growth factor, PRP = platelet-rich plasma, WOMAC = The Western Ontario and McMaster Universities (WOMAC) osteoarthritis index.

### 3.4. Patient demographics

A total of 1652 cases were collected, including 374 in the PRP group (Table 8). The average age of patients was 58.92 ± 7.05, the average BMI was 27.47 ± 3.26, and 70.85% of the patients were females. There were 616 patients in the HA group, the mean age of the patients was 58.26 ± 7.26, the mean BMI was 27.61 ± 4.03, and 77.92% of them were female. The ozone group had 150 patients in total. The mean age of the patients was 58.37 ± 6.03, the mean BMI was 27.14 ± 6.03, and 78% of the cases were female. In the CS group, there were 159 patients with an average age of 61.06 ± 5.46, an average BMI of 28.19 ± 2.9, and 48.42% of them were women. There were 219 patients in the PRGF group. The mean age of the patients was 57.25 ± 7.4, the mean BMI was 27.76 ± 4.1, and 58.9% of the patients were female. As for the HA + PRP group, there were a total of 134 patients, the mean age was 57.39 + 6.63, the mean BMI was 27.79 ± 3.78, and 66.11% of them were female.

**Table 8:** Generalities of the included studies and demographic date of the samples.

Author year	Sample size	Type of protocol	Type of injection	Sampe size	Male/Female	Age	BMI	Adverse reactions
Brain J Cole 2016^[7]^	99	Three injectionper wk	PRP	49	28/21	55.9 ± 10.4	27.4 ± 3.9	Not reported
			HA	50	20/30	56.8 ± 10.5	29.0 ± 6.4	
Seyed Ahmad Raeissadat 2018^[40]^	141	Three injectionper wk	Ozone	67	17/50	58.1 ± 6.4	26.8 ± 1.95	Not reported
			HA	74	18/56	61.1 ± 6.3	28.6 ± 1.65	
Seyed Ahmad Raeissadat 2020^[18]^	102	Three injectionper wk	PRGF	50	14/36	57.08 ± 7.3	27.92 ± 2.7	Group PRGF had 1 case of swelling, 9 cases of stiffness and heaviness at the injection site Group HA had 3 cases of stiffness and heaviness at the injection site
			HA	52	15/37	58.63 ± 7.09	28.65 ± 3.02	
Raeissadat 2021^[11]^	200	HA and Ozone (3 doses wk) PRP and PRGF (2 doses with 3 wk interval)	HA	49	13/39	56.09 ± 6.0	27.41 ± 2.6	Not reported
			PRP	52	14/37	56.07 ± 6.3	27.50 ± 2.1	
			PRGF	51	12/37	57.91 ± 6.7	27.46 ± 2.2	
			Ozone	48	12/36	57.60 ± 6.0	27.01 ± 1.9	
Askari 2016^[41]^	140	One IA injections of HA or one injection of CS.	CS	69	12/57	57.0 ± 9.1	Not reported	Not reported
			HA	71	9/62	58.5 ± 8.3		
Bijan, Forogh 2015^[42]^	39	One IA injections of PRP or one injection of CS.	PRP	23	7/17	59.13 ± 7.03	28.9 ± 2.8	Not reported
			CS	16	9/15	61.13 ± 6.7	29.2 ± 3.4	
Duymus, T M 2016^[43]^	102	PRP: 2 doses, HA a single dose Ozone 4 doses.	PRP	33	1/32	60.4 ± 5.1	27.6 ± 4.6	Not reported
			HA	34	1/33	60.3 ± 9.1	28.4 ± 3.62	
			Ozone	35	4/31	59.4 ± 5.7	27.6 ± 4.4	
Elksniņš-Finogejevs, A 2020^[33]^	36	One IA injections of PRP or one injection of CS.	PRP	19	17/3	66.5 ± 8.6	28.6 ± 5.0	Mild synovitis was registered by 15 patients (75%) in the PRP group at the first wk after treatment
			CS	17	15/5	70.1 ± 9.1	30.5 ± 5.8	
Lana, JFSD 2016^[44]^	105	3 intra-articular knee injections with 2 wk intervals between each injection	HA	36	3/33	60 ± 6.6	28.24 ± 8.77	Not reported
			PRP	36	7/29	60.9 ± 7, 6	27.42 ± 6.89	
			HA + P RP	33	6/27	62 ± 6.1	29.15 ± 7.31	
Kuan-Yu Lin 2018^[45]^	87	3 wk injections	PRP	31	9/22	61.17 ± 13.1	23.98 ± 2.62	Not reported
			HA	29	10/19	62.53 ± 9.9	26.26 ± 2.99	
			Saline Solution	27	10/17	62.23 ± 11.7	24.98 ± 3.12	
Lisi, C 2017^[46]^	54	Patients received three injections at 4-wk intervals	PRGF	29	20/10	53.5 ± 15.1	Not reported	Not reported
			HA	25	16/12	57.1 ± 10		
Sanchez, M 2012^[47]^	176	3 wk injections	PRGF	89	52/46	60.5 ± 7.9	27.9 ± 2.9	Not reported
			HA	87	52/45	58.9 ± 8.2	28.2 ± 2.7	
Ke Su 2018^[48]^	82	HA + PRP: administered twice, 2 wk apart PRP: every 14 d HA: every 7 d	HA + PRP	27	10/17	50.67 ± 8.7	28.19 ± 1.31	Not reported
			PRP	25	11/14	54.16 ± 6.5	28.17 ± 1.43	
			HA	30	12/18	53.13 ± 6.4	28.69 ± 1.13	
Uslu Güvendi, E 2018^[49]^	50	CS (receiving one c orticosteroid injection), PRP (receiving three PRP injections with one wk interval	CS	17	Not reported	62.8 ± 1.7	31.1 ± 1.0	Not reported
			PRP	33		61.3 ± 1.6	31.4 ± 0.7	
Emre, Fahri 2021^[50]^	120	15 d interval between PRP and HA injection	HA + PRP	48	45/75	59.5 ± 5	26.05 ± 2.72	Not reported
			PRP	33			25.97 ± 1.76	
			HA	39			25.82 ± 1.64	
Yong Huang 2019^[51]^	120	IA-HA (2 mL/wk, for 3 wk), IA-CS (1 mL) or IA-PRP (3 times, 4 mL, every 3 wk) groups.	HA	40	19/21	54.8 ± 1.1	24.51 ± 3.09	Not reported
			CS	40	21/19	54.3 ± 1.4	24.56 ± 3.62	
			PRP	40	25/15	53.13 ± 6.41	25.23 ± 4.15	

CS = corticosteroids, HA = hyaluronic acid, HA + PRP = hyaluronic acid and platelet-rich plasma, IA injection = intra-articular injections, PRGF = platelet-rich plasma-derived growth factor, PRP = platelet-rich plasma.

### 3.5. Adverse events

In our study, 10 cases of adverse reactions to intra-articular PRGF injection were reported, including 1 case of joint swelling and 9 cases of stiffness and heaviness after injection. In 3 cases, IA injection of HA caused joint swelling and stiffness. One week after intra-articular PRP injection, 15 patients (75%) developed mild synovitis. For the rest, no adverse reactions were reported (Table 8).

### 3.6. Publication bias analysis

**Figure 6.**
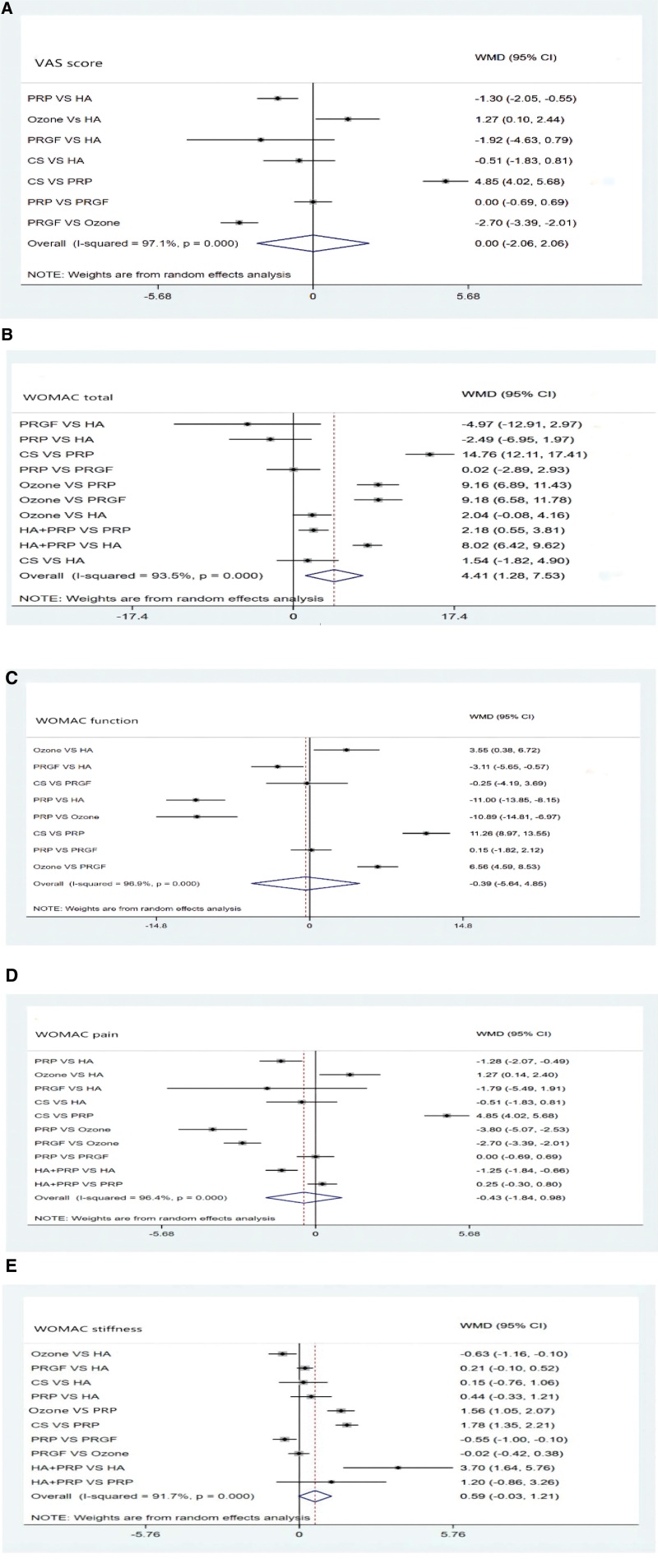
Funnel plot of publication bias (A) VAS, (B) WOMAC total, (C) WOMAC function, (D) WOMAC pain, and (E) WOMAC stiffness. VAS = visual analogue scale, WOMAC = The Western Ontario and McMaster Universities (WOMAC) osteoarthritis index.

Figure 6 shows the results of a comparison-correction funnel plot created with State (Version 15.1) software to assess the presence of publication bias. Interpretation of the findings: The points in each figure’s inverted triangle are approximately symmetrical, with the midline as the axis of symmetry, and if the correction aid is perpendicular to the angle of the midline, it indicates the presence of publication bias or small sample events.

## 4. Discussion

This network meta-analysis looked at the efficacy of various IA injection drugs that are currently used to treat mild to moderate knee osteoarthritis. Our findings suggest that, when compared to HA, CS, ozone, HA + PRP, and PRGF, intra-articular injection of PRP should be the first choice. The combination of IA injection HA + PRP has the highest chance of lowering the VAS score. Also, IA PRGF has the highest probability of lowering the WOMAC total score. We can use PRGF or PRP + HA instead of PRP for pain relief if the preparation method and economic cost are not taken into account. This is the first network meta-analysis that compares PRP, PRGF, HA + PRP, HA, CS, and ozone.

Shen et al^[11,12]^ discovered that, when compared to IA injections of HA, CS, and ozone, PRP could better reduce knee pain, lowering WOMAC scores; and, when compared to IA injections of HA and ozone, intra-articular injection of PRP could result in lower VAS and WOMAC scores. These findings are consistent with ours.

PRP has been shown in studies to repair cartilage and tendons in damaged tissue by delivering high concentrations of cytokines and GFs to the damaged tissue.^[13,14]^ Platelet-rich plasma (PRP) is created by centrifuging blood, resulting in a high concentration of platelets in a small amount of plasma. Platelet growth factors and cytokines play important roles in cell growth, angiogenesis, tissue regeneration, and other processes.^[15]^ Large amounts of growth factors and anti-inflammatory cytokines are present in PRP. Following PRP injection, growth factors like Platelet-derived Growth Factor-BB rise at various times.^[16]^ By blocking IL-1 and nuclear factor kappa-B, it also lowers the levels of proinflammatory cytokines, reducing the inflammatory effects of OA.^[1]^

It was discovered in this network meta-analysis that PRGF ranked high in optimizing the score of KOA patients, ranking first in the category of WOMAC total. In fact, PRGF, a PRP product, contains no leukocytes or inflammatory cytokines and only trace specific amount of cytokines and growth factors cytokines and growth factors.^[12]^ PRP injections may not produce enough growth factors in some cases, resulting in a poor response to treatment. To overcome this barrier, biocompatible activators were used to stimulate platelets to release their granular content, resulting in the production of PRGF.^[17]^ As a platelet-derived factor in excess of the physiological amount, PRGF can be delivered to OA sites to help cartilage heal naturally, regenerate tissue, and cause anti-inflammatory responses.^[18]^ PRGF was found to be more effective than HA in a randomized controlled multicenter trial,^[19]^ with benefits lasting 24 weeks. Another RCT ^[18]^ found that 12 months after the PRGF injection, VAS, ADL, and other indicators improved significantly compared to the baseline. Another study ^[20]^ found that PRGF and PRP injections were equally effective in OA patients, with no difference in pain relief or functional improvement. However, our research revealed that PRGF outperforms PRP in some ways, which may be due to differences in injection and preparation methods.

HA is a component of synovial fluid that serves primarily as a joint lubricant and cushion. By injecting exogenous HA into the joint, HA IA injection therapy can restore the rheological characteristics of the synovial membrane, reducing pain and improving function.^[21]^ Several clinical trial studies have demonstrated the effectiveness of HA IA injection in the treatment of KOA.^[22–25]^ However, HA injection is expensive. In addition, there is no reliable evidence that HA IA injection can effectively cure KOA.^[19]^ In addition, there is no reliable evidence that HA IA injection can effectively cure the inflammatory cascade in joints, and some patients have experienced adverse acute reactions during treatment processes.^[26–28]^According to research, combining HA and PRP has a synergistic effect on cartilage regeneration and inflammation reduction.^[29]^ Our findings confirmed that HA + PRP can significantly reduce pain in KOA patients. However, this has not been thoroughly researched, and the combined use of PRP + HA is frequently more complicated and costly.^[30]^

Intra-articular corticosteroids have been widely used in the treatment of OA for decades, and guidelines from the “Osteoarthritis Research Society International 2014” and the “American College of Rheumatology 2012” both recommend intra-articular CS for the treatment of KOA.^[31,32]^ In a study,^[33]^ both PRP and CS treatments were shown to reduce KOA symptoms in a short period of time, and while there was no difference in VAS scores, PRP was shown to be superior to CS in long-term follow-ups. However, the efficacy of CS was consistently ranked in the bottom 2 positions in this study, which could be due to the fact that the benefit time of IA injection of CS is short, so it is often effective in the short-term.^[34]^

In this study, IA ozone injection performed poorly in terms of improving the conditions of KOA patients, and the reason was thought to be the inclusion of follow up data after 3 months. After injection into the knee cavity, ozone can act directly on the joint.^[35]^ It is more soluble in blood and tissue fluids as a substance with higher oxidation and water solubility than oxygen and can exert stronger oxidative and oxygen saturation effects to improve the internal environment of the joint cavity, promote the elimination of inflammation and edema, and relieve pain.^[36]^ Early-stage effects of IA injection of ozone in the treatment of KOA were demonstrated in studies. A previous meta-analysis by Raeissadat et al^[37]^ also revealed that the effect of ozone injection on KOA gradually faded after 4 to 6 months. PRP had a longer duration of action than ozone treatment, but ozone relieved OA symptoms earlier than PRP.^[38]^ An RCT study found no difference in WOMAC score reductions by ozone and PRP after 1 month of injection, but at the 3-month follow up, the WOMAC in the ozone group was significantly higher than that in the PRP group.^[39]^ Future research must resolve disagreements about ozone long-term efficacy.

## 5. Limitations

This article searched a large number of databases, and a comprehensive collection of literature has become the network meta-analysis’s advantage. This study, however, has some limitations. First, we only looked at data from the most recent follow up visit, leaving short-term results out. Second, the interventions included in the article were insufficiently comprehensive, and other IA injection drugs were not included, resulting in a study with a limited scope.

## 6. Conclusion

To compare the long-term efficacy of several drugs, IA injection of PRP should be the first choice for the treatment of mild to moderate KOA. Intra-articular injection of HA + PRP and PRGF is better in relieving pain, but considering its expensive price or complicated preparation, it is not considered. Their short-term efficacy was not clearly demonstrated, needing more and higher-quality RCTs to show better results. In the future, it should be examined whether additional medications used for IA injections for KOA are effective.

## Acknowledgements

Many people have offered me valuable help in this writing, including my tutor and my parents. I would like to give my sincere gratitude to them.

## Author contributions

**Conceptualization:** Yuan Xue, Xiuming Wang.

**Data curation:** Yuan Xue, Xuan Wang, Aina Yao.

**Investigation:** Li Huang.

**Resources:** Yan Xue.

**Software:** Yuan Xue.

**Supervision:** Yuan Xue, Yan Xue.

**Visualization:** Yuan Xue, Aina Yao.

**Writing – original draft:** Yuan Xue.

**Writing – review & editing:** Yuan Xue.
